# A Borophosphate Glass Doped with Cobalt Oxide Improves Skeletal Muscle Structure and Function in Myopathic Mice

**DOI:** 10.3390/jfb17030155

**Published:** 2026-03-20

**Authors:** Jacob A. Kendra, Alexandra G. Naman, Rebekah L. Blatt, Carla D. Zingariello, Richard K. Brow, Steven S. Segal, Aaron B. Morton

**Affiliations:** 1 Department of Kinesiology and Sport Management, Texas A&M University, College Station, TX 77845, USA; jkendra@tamu.edu (J.A.K.); anaman@tamu.edu (A.G.N.); 2 Department of Materials Science & Engineering, Missouri University of Science & Technology, Rolla, MO 65409, USA; rlb3nf@mst.edu (R.L.B.); brow@mst.edu (R.K.B.); 3 Department of Pediatric Neurology, The Children’s Hospital at Montefiore, Bronx, NY 10467, USA; czingariel@montefiore.org; 4 Department of Medical Pharmacology and Physiology, University of Missouri, Columbia, MO 65212, USA; segalss@health.missouri.edu; 5 Dalton Cardiovascular Research Center, Columbia, MO 65211, USA; 6 Department of Pathobiology and Integrative Biomedical Sciences, University of Missouri, Columbia, MO 65211, USA; 7 Department of Biomedical, Biological and Chemical Engineering, University of Missouri, Columbia, MO 65201, USA; 8 Department of Nutrition and Exercise Physiology, University of Missouri, Columbia, MO 65211, USA

**Keywords:** angiogenesis, bioactive glass, inorganic biomaterial, myopathy, regenerative medicine, skeletal muscle

## Abstract

Skeletal muscle myopathy remains a significant cause of disability with limited treatment strategies. Advancements in tissue engineering have led to the development of borophosphate bioactive glasses (BPBGs) capable of enhancing skeletal muscle structure and function. Using a mouse model of severe myopathy (D2.*mdx*), we investigated muscle force, regeneration, angiogenesis and inflammation at 14, 70 and 140 days post-treatment (dpt). Tibialis anterior (TA) muscles of D2.*mdx* mice that received a single injection of cobalt oxide-doped BPBG (CoO-TRIM) particles exhibit greater active force, myofiber size, and regeneration through 70 dpt compared to control D2.*mdx* mice injected with Saline. Vascular endothelial growth factor (VEGF) was elevated up to 70 dpt in D2.*mdx* CoO-TRIM mice followed by increased muscle vascularity. As a marker of inflammation, interleukin (IL)-6 increased in D2.*mdx* CoO-TRIM mice compared to D2.*mdx* Saline controls at 14 dpt, with no differences at 70 or 140 dpt. No differences were observed in outcome measures between wild-type (WT) CoO-TRIM mice and WT Saline controls. We report that CoO-TRIM can stimulate VEGF production and promote restoration of muscle structure and function when inflammation is present. Local injection of an inorganic biomaterial alone can benefit myopathic skeletal muscle.

## 1. Introduction

Skeletal muscle myopathies are a heterogeneous group of disorders characterized by impaired muscle structure and function, which lead to progressive weakness and diminished myofiber integrity resulting in frequent injury and chronic tissue damage [1,2,3]. These conditions arise from diverse etiologies, including genetic mutations [4,5], persistent inflammatory and autoimmune dysfunction [3,6], and metabolic dysregulation [7,8]. Multiple myopathic conditions exhibit common pathological phenotypes, including reduced force production [9,10], smaller myofiber size [11,12], ischemia [13,14,15], and increased proteolytic activity [16,17]. As myopathic skeletal muscle undergoes chronic cycles of degeneration and regeneration in response to tissue damage, cellular repair mechanisms are depleted, culminating in progressive wasting and eventual replacement with non-contractile tissue through increased activity of fibroblasts and adipocytes [18,19,20]. Despite advances in standard care practices, effective disease-modifying therapies remain limited for many forms of myopathy.

Bioactive glasses are inorganic, non-crystalline, third-generation biomaterials, capable of temporal release of incorporated ions stimulating specific cellular responses for regeneration of living tissue [21,22,23,24]. While organic biomaterials (hydrogels, collagen matrices, etc.) have been used in the context of soft tissue (muscle, vasculature, nerves) repair, inorganic biomaterials have been used for hard tissue applications. Examples include particles in toothpaste to restore enamel of sensitive teeth [25], as scaffolds to repair bone defects [22], and as surface coatings for implanted biomedical devices [26]. Historically, the gold standard for biocompatible inorganic material was silica-based (SiO_2_) 45S5 (Bioglass^®^), the original bioactive glass designed to form a hydroxyapatite layer, bonding the scaffold to tissue structure [22]. However, limitations for 45S5 use in soft tissue include incomplete degradation, with ~85% of particles remaining 14 days after incubation in simulated body fluid (SBF) [27,28]. Furthermore, early stages of 45S5 dissolution trigger pH-dependent cytotoxicity: accelerated rates of ion exchange in aqueous solutions result in a burst of alkaline ion release that alters the pH of local microenvironments [29,30].

Given the limitations of early inorganic compositions based on silicates [31,32], interest in borate-based bioactive glasses has grown due to their rapid degradation and support of cellular proliferation in soft tissue [33,34,35]. In a rat model of volumetric muscle loss (VML), implantation of a borate-based bioactive glass promoted angiogenesis, improved myofiber regeneration, and increased VEGF expression [36]. Such findings point to borophosphate bioactive glasses (BPBGs) as ideal candidates for soft-tissue healing through controlled degradation while maintaining pH neutrality [35,37,38,39]. In addition, cobalt oxide (CoO)-doped glasses have emerged in soft-tissue healing for their pro-angiogenic effects, stimulating VEGF secretion and enhanced angiogenesis, effectively restoring the microvasculature to the site of injury [40,41,42,43,44]. Moreover, CoO influences macrophage extracellular vesicle secretion containing modulators of angiogenesis, including VEGF and nitric oxide synthase [45]. Cobalt’s inhibition of hydroxyapatite formation offers complementary advantages for use in skeletal muscle [40,46]. However, it is unknown how the injection of a BPBG doped with cobalt affects angiogenesis, muscle structure, and function in myopathic skeletal muscle.

In the present study, we evaluate muscle structure and function following local injection of a CoO-doped inorganic BPBG [CoO-Time Release Ion Matrix (CoO-TRIM)] into the tibialis anterior (TA) muscle of a mouse exhibiting severe skeletal muscle myopathy (D2.*mdx* mice). CoO-TRIM is formulated to release ions associated with angiogenesis and myogenesis in vivo while maintaining a neutral pH. It is prepared as a lyophilized powder and suspended in Saline prior to injection within the designated myofascial compartments. The present experiments focused on two questions using an established mouse model of severe myopathy: (1) Is skeletal muscle structure and function in D2.*mdx* mice improved following treatment with CoO-TRIM? (2) How persistent are the improvements in this model of myopathy? Quantitative physiological, histological, and biochemical analyses of the TA muscle were applied to evaluate tissue structure and function following intramuscular injection of CoO-TRIM. Experiment 1 determined whether reversal of a myopathic phenotype occurs 14 days post-treatment (dpt) following CoO-TRIM injection into the TA muscle. Experiment 2 determined whether improvements in muscles treated with CoO-TRIM persist at 70 and 140 dpt.

## 2. Materials and Methods

### 2.1. Biomaterial Fabrication

CoO-TRIM is a CoO-doped, Na-Ca–borophosphate glass with an analyzed composition (% mole): 40.5% B_2_O_3_, 18.3% P_2_O_5_, 21.8% CaO, 14.4% Na_2_O, 4.0% CoO (Table 1). Glasses were prepared from mixtures of reagent grade NaPO_3_ (Shanghai Muhong Industrial Co., Ltd., Optical Grade, Shanghai, China), Ca (PO_3_)_2_ (Shanghai Muhong Industrial Co., Ltd., Optical Grade, Shanghai, China), H_3_BO_3_ (Fisher, ≥99.5%, Waltham, MA, USA), Na_2_CO_3_ (Alfa Aesar, 99.5%, Ward Hill, MA, USA), CaCO_3_ (Fisher, >98.0%, Waltham, MA, USA), CoO (Sigma Aldrich, 99.5%, St. Louis, MO, USA), and Eu_2_O_3_ (Research Chemicals, Phoenix, Arizona, USA, 99.9%) as raw materials. The fluorescent europium (Eu)-TRIM has the same base composition as the CoO-TRIM except that CoO was replaced with 1.0 wt% Eu_2_O_3_ to provide visual evidence for particle diffusion following injection into a muscle compartment. The glasses were prepared using conventional processing methods. Briefly, raw materials were mixed by hand, placed in a platinum crucible and calcined overnight at 300 °C to evolve water and CO_2_, then melted at 1150 °C for 30 min, stirred with a platinum rod, and then melted for another 30 min. The homogeneous melt was poured into graphite molds, then annealed at 350 °C for one hour before cooling to room temperature. Annealed glasses were ground to form the CoO-TRIM particles (diameter, <10 microns) using a Spex mill and stored in 1.5 mL microcentrifuge tubes in a desiccator prior to use.

### 2.2. Biomaterial Dissolution Kinetics

Dissolution experiments were conducted using a single-pass flow through (SPFT) system with Kokubo’s SBF, prepared as described [47]. CoO-TRIM powder (200 mg) was placed between 0.45 µm filter papers in a sample cassette mounted in a 37 °C oven. A syringe pump drove a continuous flow of SBF through the filter at 1 mL/h. Effluent solution was collected every 12 h for the first 4 days, then every 24 h for the next 10 days. After 14 d, the powder was removed and rinsed with ethanol, dried in a 70 °C oven, and then placed in a desiccator prior to testing. Effluent solution pH was recorded at room temperature. Ion concentrations in the effluent solution were analyzed using Inductively Coupled Plasma–Optical Emission Spectroscopy (ICP-OES) using a Perkin-Elmer Avio 200 (PerkinElmer, Inc; Waltham, MA, USA). The means and standard error of the mean (SEM) of the room temperature solution pH and ion concentrations at each time point are reported. A second batch of TRIM particles, before and after a 7 d SPFT experiment, were dissolved in nitric acid and their compositions were analyzed by ICP-OES. These TRIM particles were dissolved overnight in a pH = 10 solution containing 5 mM EDTA and 0.22 M NaCl and their phosphate anion distributions were then analyzed by high-performance liquid chromatography (HLPC). HPLC analysis was performed in triplicate with a Dionex GP50-2 pump (Dionex; Sunnyvale, CA, USA), an Ionpack AS7 4 × 250 mm analytical ion exchange column (Dionex; Sunnyvale, CA, USA), and an AD25 absorbance detector (Dionex; Sunnyvale, CA, USA) as described [48]. X-ray diffraction (XRD) analyses of the reacted particles were done with a PANalytical X’Pert Multipurpose diffractometer (Malvern; Worcestershire, United Kingdom) with a Cu Kα source and a PIXcel detector. To demonstrate the in vivo distribution of particles throughout the anterior compartment of the lower leg (TA and EDL), Saline and EU-TRIM particles resuspended in Saline were injected into the TA of a C57BL/6J mouse (RRID: IMSR_JAX:000664, Strain 000664, Jackson Labs; Bar Harbor, ME, USA). The mouse was then euthanized via anesthetic overdose and cervical dislocation. TA and EDL muscles from both hindlimbs were removed and illuminated with a UV light to excite EU particles within the muscles.

### 2.3. Ethical Approval and Animals

All protocols in this study were approved by Animal Care and Use Committees at the University of Missouri (AUP 17720) and Texas A&M University (AUP 2022-0215). Animal care was in accordance with the National Research Council’s Guide for the Care and Use of Laboratory Animals (Eighth Edition, 2011). Male D2.B10-DMD*^mdx^*/J (D2.*mdx*) (*n* = 5–8/group at each timepoint; RRID: IMSR_JAX:013141, Strain 013141, Jackson Labs; Bar Harbor, ME, USA) and male C57BL/6J (WT) (*n* = 4–5/group at 14-day timepoint; RRID: IMSR_JAX:000664, Strain 000664, Jackson Labs; Bar Harbor, ME, USA) were housed in the University of Missouri or Texas A&M University animal care facilities and studied at 5–7 months of age. Male 7-month-old D2.*mdx* mice were selected as the most appropriate mouse model to recapitulate human characteristics of severe skeletal muscle myopathy based on the severity of progressive limb muscle weakness, reduced myofiber size, impaired regeneration, chronic inflammation and fibrotic tissue deposition [49,50,51]. While the D2.*mdx* mouse is a model of Duchenne muscular dystrophy, the observed myopathic phenotypes beyond 6 months of age are similar to myopathies including cachexia, sarcopenia, and ischemia. Healthy C57BL/6J mice were selected as the most appropriate WT control for CoO-TRIM treatment of myopathy because they do not exhibit naturally occurring inflammation observed in the DBA/2J background strain [52,53,54,55,56], which may confound our results as previous work suggests the effects of CoO-TRIM may be immune cell-mediated [57]. Mice were housed on a 12 h:12 h light: dark cycle at ~23 °C, with fresh water and food available ad libitum.

### 2.4. Experimental Design

#### 2.4.1. Experiment 1: Are Myopathic Skeletal Muscle Structure and Function Improved Following CoO-TRIM Treatment

**Figure 1:**
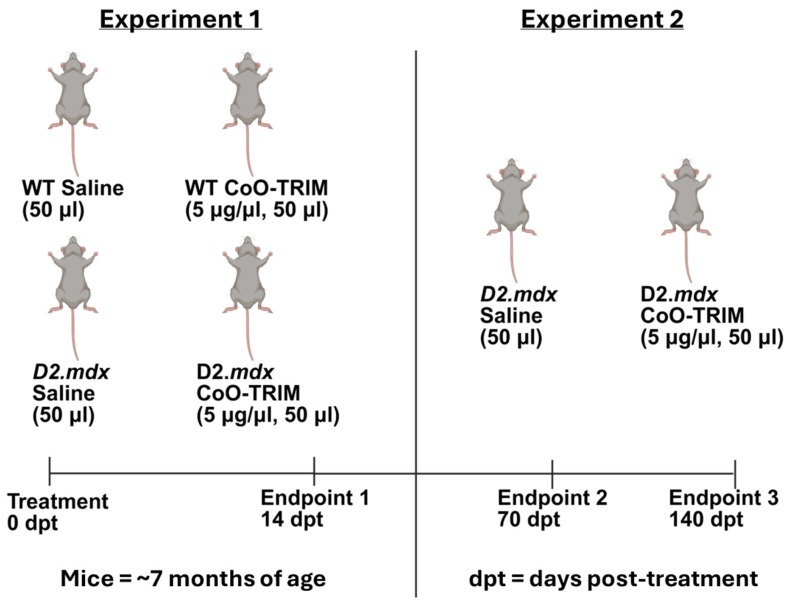
Experiment 1 evaluated myopathic muscle structure and function following intramuscular injection of Saline or CoO-TRIM in WT and D2.*mdx* mouse TA muscles 14 dpt. To evaluate how persistent improvements in myopathic muscle structure and function are following intramuscular injection of CoO-TRIM, experiment 2 evaluated only D2.*mdx* mouse TA muscles at 70 and 140-dpt.

These experiments evaluated WT and D2.*mdx* mice at 14 dpt by comparing responses to injection of Saline alone or with CoO-TRIM (Figure 1). Mice were randomly assigned to one of the following experimental groups: (1) healthy vehicle alone (WT Saline); (2) healthy CoO-TRIM (WT TRIM); (3) myopathic vehicle alone (D2.*mdx* Saline); (4) myopathic CoO-TRIM (D2.*mdx* TRIM). A mouse was anesthetized with ketamine and xylazine (100 mg/kg and 10 mg/kg, respectively; intraperitoneal injection) or isoflurane inhalation (4% induction, 2% maintenance, 100% Oxygen) and 50 µL of Saline or 250 µg of CoO-TRIM suspended in 50 µL of Saline (5 µg/µL) was injected into the TA muscle of both hindlimbs. To determine treatment dosage, a preliminary dose–response study was conducted at 14 dpt comparing 2.5 µg CoO-TRIM/µL (0.5× dose), 5 µg CoO-TRIM/µL (1× dose), and 10 µg/µL CoO-TRIM (2× dose). Muscle force during isometric contractions following the 0.5× dose was unchanged compared to untreated control (CON) (0.5× dose 1.70 g vs. CON 1.69 g of force/mg TA weight), and the 2× dose did not significantly improve force above the effect of the 1× dose (2× dose 1.88 g vs. 1× dose 1.87 g of force/mg TA weight). Therefore, the present experiments here used the 1× dose (5 μg CoO-TRIM per muscle). On the day of an experiment, the left TA muscle was prepared for in situ measurements of maximal (max) isometric force as the criterion for muscle function [58,59]. The mouse was then euthanized via anesthetic overdose and cervical dislocation. TA muscles from both hindlimbs were removed and stored at −80 °C for histological and biochemical analyses.

#### 2.4.2. Experiment 2: How Persistent Are Improvements of Myopathic Muscle Structure and Function Following CoO-TRIM Treatment

These experiments evaluated only D2.*mdx* mice at 70 and 140 dpt with Saline alone or with CoO-TRIM injected as in experiment 1 (Figure 1). On the day of an experiment, the left TA muscle was prepared for in situ measurements of maximal (max) isometric force and euthanized for tissue harvest as described above. Because there were no differences in maximal force, myofiber size, CD31 immunostaining, VEGF concentration, and inflammatory cytokine (CXCL1, IL-6, TNFα, IL-1β, IL-10, IFNγ, IL-12p70, IL-2, IL-5) concentrations of WT mouse groups following injection of Saline alone or with CoO-TRIM in experiment 1, only D2.*mdx* mouse groups were studied at 70 and 140 dpt.

### 2.5. Evaluation of Maximal Isometric Force

The mouse was anesthetized (as above) and weighed. A 2-0 silk suture was secured around the left patellar tendon. The distal tendon of the TA muscle was isolated, secured with 2-0 suture, and then severed from its insertion. The mouse was placed prone on a heating platform positioned on a plexiglass board, and the patellar tendon was tied to a vertical metal peg with 2-0 silk suture to immobilize the knee. The TA tendon was tied to a load beam (LCL-113G; Omega, Stamford, CT, USA) coupled to a Transbridge amplifier (TBM-4; World Precision Instruments, Sarasota, FL, USA). The load beam was attached to a micrometer to adjust the optimal length (L_o_), as determined during twitch contractions at 1 Hz. A strip of KimWipe^®^ was wrapped around the exposed TA muscle and irrigated (3 mL/min) with bicarbonate-buffered physiological salt solution (bbPSS) warmed to 40 °C; a heat lamp maintained the muscle at 32 °C. Monophasic pulses (0.1 ms, 10 V) were delivered from an S48 stimulator coupled to a SIU5 stimulation isolation unit (Grass Instruments, West Warwick, RI, USA). For direct field stimulation, a pair of platinum/iridium (90%/10%) wire electrodes (diameter, 250 μm) was placed across the muscle belly; a Stimu-Splitter II (Med-Lab Instruments, Loveland, CO, USA) was coupled to a second Grass S48 stimulator (Grass Instruments, West Warwick, RI, USA) to generate sufficient current to depolarize myofibers. Based on preliminary experiments to define the frequency–force relationship of the TA muscle in situ, maximum tetanic contractions were obtained at 120 Hz for 500 ms. Data for force production were acquired using Power Lab software (v8.1.31, ADInstruments, Colorado Springs, CO, USA) on a personal computer. Following contractions, the TA muscle was excised, blotted of excess moisture, and weighed (XP205 Pro, Mettler Toledo; Columbus, OH, USA).

### 2.6. Immunohistochemistry and Histology Analysis

Primary antibodies used were rat anti-CD31 (1:200, RRID: AB_393571, BD Biosciences, Franklin Lakes, NJ, USA) and rabbit anti-laminin (1:400, Cat.# NC1732938, Fisher Scientific; Hampton, NJ, USA). Secondary antibodies were all from Fisher Scientific (Hampton, NJ, USA): Alexa Fluor 647 Goat anti-rat (1:400, RRID: AB_2895299, Cat.# A48265) and Goat anti-rabbit Rhodamine (TRITC) (1:400, RRID: AB_90296, Cat.# AP132RMI).

Left TA muscles were embedded in Tissue-Plus™ O.C.T. Compound (Scigen, Fischer Scientific, Hampton, NJ, USA), frozen in liquid nitrogen-cooled 2-methyl butane (Thermo Fisher Scientific, Waltham, MA, USA) and sectioned (thickness, 10 μm) in a Cryostar NX50 Cryostat (Epredia, Kalamazoo, MI, USA) at −17 °C onto a microscope slide. Sections were fixed with ice-cold 4% paraformaldehyde for 20 min, washed 3× in Tris-buffered Saline (TBS), then permeabilized in 0.5% Triton X-100. Primary antibodies were incubated for 60 min at room temperature (RT) in blocking buffer (Pierce™ Protein-Free Blocking Buffer, Thermo Fisher Scientific; Waltham, MA, USA). Sections were washed 3× in TBS, incubated with secondary antibodies in blocking buffer for 60 min at RT, washed 3× in TBS, and mounted in Invitrogen™ ProLong™ Gold antifade reagent with DAPI (Cat.# P36941, Fisher Scientific, Hampton, NJ, USA). Slides were imaged on a Stellaris 5 White Light Laser confocal microscope (Leica Microsystems, Deer Park, IL, USA) using Leica LAS_X software (v4.5.0.25531, RRID: SCR_013673, Leica Microsystems, Deer Park, IL, USA).

For myofiber cross-sectional area (CSA) and CD31^+^ analysis, 3–5 randomized 580 µm × 580 µm regions of interest (ROI) spanning the entire muscle were imaged with 10× (N.A., 0.40) or 20× (N.A., 0.75) objectives and values across all regions were averaged per muscle. An average of over 400 myofibers per TA muscle section were evaluated. Myofiber CSA was analyzed via semi-automatic muscle segmentation analysis in NIS-Elements Advanced Research software (v5.42.06, RRID: SCR_014329, Nikon, Melville, NY, USA) presented as µm^2^. Myofibers were classified as small myofibers (<800 µm^2^) or large myofibers (>2000 µm^2^). The total area of CD31^+^ staining of endothelial cells (ECs) was measured and normalized to the number of myofibers in each ROI to evaluate microvascular area using Aivia machine learning software (v10.5, Leica LAS_X Software imported calibration: 0.56 µm/px) (Aivia, Bellevue, WA, USA). Additionally, the number of CD31^+^ puncta was divided by the number of myofibers for the EC-to-myofiber ratio in each ROI. For centrally located nuclei (CLN) analysis, 10× (N.A., 0.40) tile square images were acquired and merged in Leica LAS_X software (RRID: SCR_013673, Leica Microsystems, Deer Park, IL, USA). Using NIH ImageJ (v1.54d, RRID: SCR_003070, NIH, Bethesda, MD, USA), the total number of CLN^+^ myofibers was counted and normalized to the total area (mm^2^) of the muscle cross-section as an index of myofiber regeneration. Furthermore, the total number of myofibers (CLN^+^ + CLN^−^) was counted and normalized to the total area (mm^2^) of the muscle cross-section to assess myofiber count.

For fibrotic-tissue analysis in D2.*mdx* TA muscle, 10 μm sections were permeabilized in 0.5% Triton X-100 in TBS and incubated for 60 min with PicroSirius Red (0.5 g Direct Red in 500 mL saturated picric acid). Thereafter, TA muscle cross-sections were treated for 30 min with 0.5% acetic acid in ddH_2_O and rinsed in 100% ethanol. Images were acquired with a 4× objective (N.A., 0.13) on a Nikon E800 microscope coupled to a DS-Fi3 camera using NIS-Elements Advanced Research software (RRID: SCR_014329, Nikon, Melville, NY, USA). Using NIH ImageJ, images were converted to binary, and the total area occupied by connective tissue was divided by the area of the muscle cross-section, multiplied by 100 for the % fibrotic area.

### 2.7. Muscle Tissue Preparation

Right TA muscle samples from WT TRIM, WT Saline, D2.*mdx* TRIM, and D2.*mdx* Saline mice at 14 dpt, and then D2.*mdx* TRIM, and D2.*mdx* Saline mice at 70 and 140-dpt, were homogenized in lysis buffer (pH = 7.4) containing 5 mM Tris-HCI, 5 mM EDTA with protease and phosphatase inhibitors (Sigma-Aldrich, St. Louis, MO, USA). Homogenates were centrifuged at 1500× *g* for 10 min at 4 °C. The supernatant (soluble fraction) was aspirated, and its protein concentration assessed using the Bradford method (Sigma-Aldrich, St. Louis, MO, USA). TA muscle tissue supernatant was used for Western blot and enzyme-linked immunosorbent assay (ELISA) analysis.

### 2.8. Western Blot Analysis

Protein concentration of each sample was normalized in 4× Laemmli sample buffer (Cat. # 1610747, Bio-Rad, Hercules, CA, USA) containing 5% dithiothreitol. Samples were loaded on 4–20% gradient Criterion TGX Midi-Protein gels (Bio-Rad, Hercules, CA, USA) for electrophoresis and transferred to LF-PVDF membranes (Millipore, Burlington, MA, USA). Following transfer, membranes were blocked in either 5% milk solution or Intercept TBS Blocking Buffer (Li-Cor Biotechnology, Lincoln, NE, USA) for 1 h at room temperature; followed by incubation with primary antibodies overnight at 4 °C. Membranes were exposed to either IRDye 800CW Goat anti-Mouse IgG (RRID: AB_621842, Cat.# 926-32210) or IRDye 800CW Goat anti-Rabbit IgG secondary antibodies (RRID: AB_621843, Cat.# 926-32211) (Li-Cor Biotechnology, Lincoln, NE, USA) for 30–60 min depending on the protein of interest.

Membranes were scanned with a Li-Cor Odyssey DLx Imager (Li-Cor Biotechnology, Lincoln, NE, USA) and analyzed using Image Studio Lite. Primary antibodies of interest were; Alpha II spectrin (RRID: AB_2194351, Cat.# sc-48382; Santa Cruz Biotechnology, Santa Cruz, CA, USA, 1:250 incubated overnight at 4 °C then 3 h at room temperature secondary concentration 1:5000) and LC3B (RRID: AB_915950, Cat.# 2775; Cell Signaling Technology, Danvers, MA, USA, secondary concentration 1:5000). Western blots were normalized to total protein according to recommendations for fluorescent Western blotting [60] using Revert total protein stain (Cat.# 926-11011, Li-Cor Biotechnology, Lincoln, NE, USA). Note, the 40 kDa bands correlate with the total protein in each lane and are shown only as a representative image (for space limitations) of the protein loading. Individual data points are displayed as densitometric values normalized to total protein expression (A.U., arbitrary units). Full total protein images used to normalize individual proteins are available in the data supplement.

### 2.9. ELISA

Samples of muscle supernatant (50 µL; referred to above) were analyzed to determine VEGFA protein concentration using a Quantikine ELISA (Cat.# MMV00, R&D Systems, Minneapolis, MN, USA) according to the manufacturer’s instructions. Given limited tissue availability and that no differences were observed in any other measure between 14 dpt WT Saline and TRIM-treated, and 11 dpt WT Saline and TRIM-treated, all ELISA experiments were performed on 11 dpt WT controls. Concentrations were determined by comparing samples to recombinant VEGFA protein standard supplied by the manufacturer and normalized to individual sample protein concentrations assessed by the Bradford method as mentioned above. Muscle supernatant (50 µL) was also analyzed to determine cytokine and chemokine concentrations using a V-Plex Proinflammatory Panel 1 (mouse) kit (Cat.# K15048D-1, Meso Scale Discovery, Rockville, MD, USA) according to the manufacturer’s instructions. The kits measured cytokine levels for the following: IFN-y, IL-1B, IL-2, IL-4, IL-5, IL-6, IL-10, IL-12p70, KC/GRO (CXCL1), and TNF-a. Concentrations were determined by comparing samples to the recombinant protein standards of each cytokine/chemokine supplied by the manufacturer and normalized to individual sample protein concentrations as above.

### 2.10. Statistical Analysis

Summary data are reported as means ± SEM. Statistical analyses were performed using Prism 9 software (v10.6, RRID: SCR_002798, GraphPad Software, La Jolla, CA, USA). Data from experiment 1 was analyzed using two-way ANOVA (Disease × treatment) with Sidak’s multiple comparisons. Data from experiment 2 were analyzed using Student’s *t*-test to determine statistical significance among group mean differences when appropriate. Values for “*n*” refer to the number of mice studied in each experimental group. *p* ≤ 0.05 was considered statistically significant. A power analysis was conducted a priori on previous muscle force experiments at 14 days, indicating *n* = 5 was sufficient to reach a power of 0.8 [57]. Additional assays were checked for power post hoc.

## 3. Results

### 3.1. CoO-TRIM Properties

**Figure 2:**
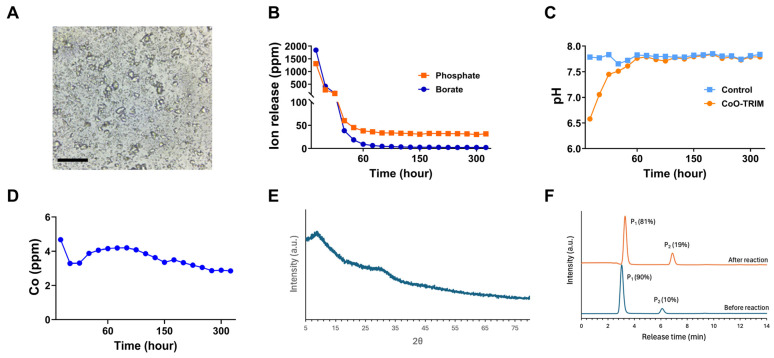
Dissolution and conversion of TRIM in the single-pass flow through (SPFT) test with 37 °C simulated body fluid (SBF). (**A**) Representative optical image of TRIM particles suspended in SBF, Scale bar = 10 µm. (**B**) Normalized release kinetics of borate and phosphate species across 14 days (324 h). (**C**) SBF pH across 14 days in the SPFT test. (**D**) Cobalt ion release profile across 14 days. (**E**) X-ray diffraction shows that the reacted TRIM particles are X-ray amorphous. (**F**) High-performance liquid chromatographs (HPLCs) of the TRIM particles before and after the SBF SPFT test. TRIM converts to an amorphous calcium polyphosphate phase composed of orthophosphate (P_1_) and pyrophosphate (P_2_) anions.

Figure 2A shows an optical image of the CoO-TRIM particles in SBF. Supplementary Figure S1 shows the fluorescent image of the EU-TRIM particles that have diffused through the anterior compartment of the lower-leg (TA and EDL) muscles following a single injection into the TA muscle. Figure 2B,C summarize the changes in pH and the ion release data, respectively, from CoO-TRIM particles during the dynamic 14 d SPFT tests with 37 °C SBF across the indicated time points. The CoO-TRIM particles release 100% of their original borate content and 50% of their original phosphate content in the first 3 days of the dynamic SPFT tests with SBF (Figure 2B). Here, the borate and phosphate concentrations were normalized by subtracting the respective species concentrations in an SBF control stream that passed through an empty sample cassette during the SPFT tests, and then normalizing those species concentrations to their total respective concentrations in the initial TRIM samples. The SBF pH, measured at room temperature, became slightly acidic during the initial reactions (Figure 2C), in contrast to the typical, alkaline TRIMs [37]. Co-ions were released initially at a lower concentration, but then at a constant concentration (4 PPM) (Figure 2D) capable of stimulating regeneration and angiogenesis [41,42,61]. Cobalt, while cytotoxic in excess, is a known stimulator of angiogenesis [45,62,63] and can inhibit hydroxyapatite (HA) formation [46,64,65].

Table 1 shows the molar concentrations of TRIM particles before and after a 7 d dynamic SPFT test with SBF. The glasses react by releasing borate and sodium ions, then precipitating a Co-doped Ca-Mg-phosphate phase. (Mg^2+^ is a component of the SBF.) Figure 2E shows that the reacted TRIM particles are X-ray amorphous. Figure 2F shows the HPLC results for TRIM particles, before and after the 7 d SPFT test. Here, the first peak (P_1_) in each chromatograph is due to orthophosphate anions released from the dissolved particles and the second peak (P_2_) is due to pyrophosphate anions. The reacted particles contain nearly twice the concentration of pyrophosphate anions (P_2_, 19% of the total phosphate) compared to the unreacted TRIM particles (10% of the total phosphate). Pyrophosphate anions are also known to inhibit HA formation [66,67]. The conversion of the TRIM particles into an amorphous calcium polyphosphate precipitant is similar to what has been reported previously for borophosphate glasses after reaction in SBF [37].

### 3.2. Experiment 1

#### 3.2.1. Muscle Function and Structure Is Improved Following CoO-TRIM Injection

**Figure 3:**
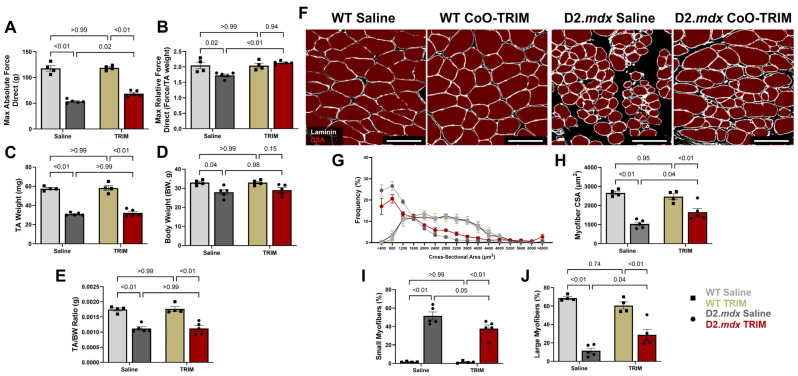
TA muscles from D2.*mdx* CoO-TRIM-treated mice exhibit greater max force and improved myofiber size. Summary values for (**A**) max absolute force and (**B**) max relative force via direct muscle stimulation at 120 Hz at 14 dpt (*n* = 4–5/group). Summary values for (**C**) TA weight, (**D**) body weight, and (**E**) muscle-to-body weight ratio at 14 dpt (*n* = 4–5/group). (**F**) Representative images of TA muscle cross-sections at 14 dpt. Laminin (white) identifies basal laminae surrounding myofibers (red), which were quantified for cross-sectional area (CSA). (**G**) Myofiber CSA frequency distribution; TRIM increases mean myofiber CSA, frequency (%) of large myofibers (CSA > 2000 μm^2^), and reduces small myofibers (CSA < 800 μm^2^). Summary values comparing (**H**) mean myofiber CSA: (**I**) small and (**J**) large myofiber frequency at 14 dpt (*n* = 4–5/group). Summary values are means ± SEM. Comparisons made vs. WT and vehicle controls by 2-Way ANOVA; *p* < 0.05 = significant. Scale bars = 100 µm.

At 14 dpt, TA muscles of D2.*mdx* CoO-TRIM mice increased maximal absolute force (g; 28%, *p* = 0.02) and relative force (max force normalized to TA weight; 23%, *p* ≤ 0.01) compared to TA muscles of D2.*mdx* Saline mice. D2.*mdx* mouse groups experienced over 50% decreased maximal absolute force compared to WT mouse groups. TA muscles of D2.*mdx* Saline mice decreased in relative force (*p* = 0.02) while TA muscles of D2.*mdx* CoO-TRIM mice had similar relative force (*p* = 0.94) compared to WT mouse groups, respectively. In contrast, there were no differences in absolute force (*p* ≥ 0.99) or relative force (*p* ≥ 0.99) between TA muscles of WT mouse groups (Figure 3A,B). D2.*mdx* mouse groups had reduced TA muscle weight (mg) and TA-to-body weight (BW) ratio compared to WT mice groups (Figure 3C–E). No change was observed in TA muscle weight or TA-to-BW ratio between WT mouse groups.

Myofiber CSA was partitioned into 400 µm^2^ increments to create relative frequency histogram plots. CSA frequency curves were shifted to the right for TA muscles of D2.*mdx* CoO-TRIM mice (Figure 3F,G) due to a ~27% reduction in small myofibers (<800 µm^2^, *p* = 0.05) and a ~17% increase in large myofibers (>2000 µm^2^, *p* = 0.04) compared to TA muscles of D2.*mdx* Saline mice (Figure 3I,J). TA muscles of D2.*mdx* CoO-TRIM mice had a 60% increase in mean myofiber CSA (*p* = 0.04) compared to TA muscles of D2.*mdx* Saline mice (Figure 3H). TA muscles of D2.*mdx* mouse groups had decreased myofiber CSA (*p* ≤ 0.01) compared to TA muscles of WT mouse groups. No change was observed in mean myofiber CSA between TA muscles of WT mouse groups (*p* = 0.95). No change was observed in fibrotic tissue deposition (*p* = 0.43) between TA muscles of D2.*mdx* groups (Supplementary Figure S2).

**Figure 4:**
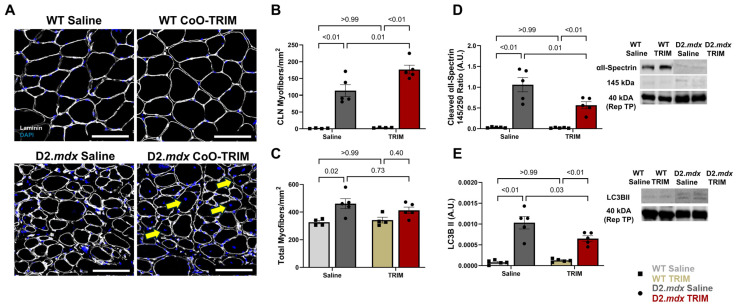
CoO-TRIM increased myofibers with centrally nucleated myofibers (CLN) and modulated proteolytic activity. (**A**) Representative images of TA muscle cross-sections at 14 dpt. Laminin (white): basal laminae; DAPI (blue): nuclei. Yellow arrows depicting position of central nuclei. (**B**) Summary values (*n* = 4–5/group) for total myofibers with CLN normalized to TA muscle cross-section area (mm^2^) at 14 dpt. (**C**) Summary values (*n* = 4–5/group) for total myofibers (CLN^+^ + CLN^−^) normalized to TA muscle cross-section (mm^2^) at 14 dpt. (**D**) Representative immunoblot for αII-Spectrin. The 145 kDa cleavage byproduct was normalized to total protein per lane, represented by the 40 kDa band from the total protein stain, and analyzed as a ratio of the 250 kDa band of αII-Spectrin. Mean densitometric data revealed a significant reduction in cleaved αII-Spectrin abundance in TA muscles of D2.*mdx* TRIM mice at 14 dpt. (**E**) Representative immunoblot of LC3B II and mean densitometric data at 14 dpt revealed reduced autophagosome number following CoO-TRIM administration in D2.*mdx* mice. (*n* = 5/group); Summary values are means ± SEM. Comparisons made vs. WT and vehicle controls by 2-Way ANOVA, *p* < 0.05 = significant. Scale bars = 100 µm.

Nuclei were counterstained with DAPI, and the total number of CLN^+^ myofibers was normalized to the total area (mm^2^) of the muscle cross-section as an index of myofiber regeneration. CLN^+^ myofibers in TA muscles of D2.*mdx* CoO-TRIM mice increased by 55% (*p* = 0.01) compared to TA muscles of D2.*mdx* Saline mice (Figure 4A,B), suggesting that CoO-TRIM may restore the regenerative capacity of damaged myofibers in TA muscles of D2.*mdx* mice [20]. Similar results were observed when CLN^+^ myofibers were analyzed as a percentage of total myofibers (Supplementary Figure S3). Accounting for total myofiber count (CLN^+^ + CLN^−^) normalized to the total area (mm^2^) of the muscle cross-section, TA muscles of D2.*mdx* Saline mice increased myofiber number (*p* = 0.02) compared to TA muscles of WT mouse groups (Figure 4C). There were no differences in myofiber number for TA muscles of D2.*mdx* CoO-TRIM mice compared to TA muscles of D2.*mdx* Saline mice (*p* = 0.73) or TA muscles of WT mouse groups (*p* = 0.40) (Figure 4C). No differences were observed in myofiber regeneration (*p* ≥ 0.99) or total myofiber number (*p* ≥ 0.99) between TA muscles of WT mouse groups.

#### 3.2.2. Muscle Proteolysis Is Reduced Following CoO-TRIM Injection

Calpain activation contributes to the proteolysis of myopathic muscle [16,68,69]. Active calpain 1 & 2 cleaves the skeletal muscle structural protein αII-spectrin from 250 to a 145 kDa product. Therefore, we measured calpain activity by Western immunoblotting for αII-spectrin 145 kDa cleaved-protein byproduct. Densitometric values were normalized to total protein and the cleaved 145 kDa byproduct was analyzed relative to the 250 kDa band. αII-spectrin cleavage byproduct indicative of calpain activity in TA muscles of D2.*mdx* CoO-TRIM mice reduced 14 dpt (*p* = 0.01) compared to TA muscles of D2.*mdx* Saline mice (Figure 4D). Similarly, LC3BII, an indicator of autophagosome formation [70], normalized to total protein in TA muscles of D2.*mdx* CoO-TRIM mice decreased (*p* = 0.03) (Figure 4E) compared to TA muscles of D2.*mdx* Saline mice. While TA muscles of D2.*mdx* mice had increased αII-spectrin cleavage byproduct indicative of calpain activity (*p* ≤ 0.01) and LC3BII (*p* ≤ 0.01) compared to TA muscles of WT mouse groups, there were no differences between WT groups (*p* ≥ 0.99).

#### 3.2.3. Angiogenesis Is Increased Following CoO-TRIM Injection

**Figure 5:**
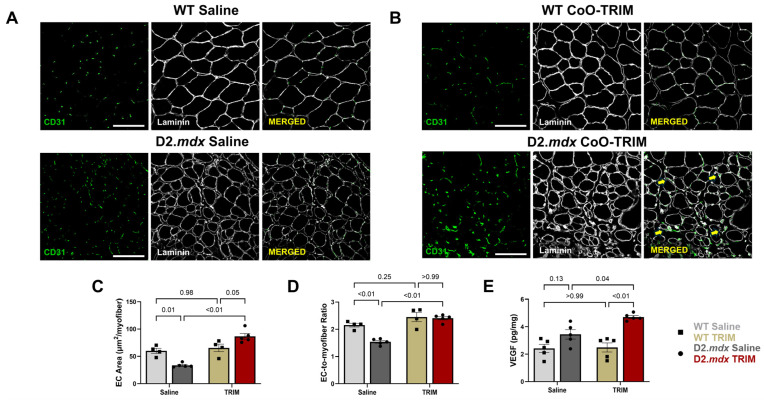
TRIM enhances vascularity. (**A**,**B**) Representative images of TA cross-sections in WT and D2.*mdx* mice at 14 dpt. Laminin + immunostaining (white) identified basal laminae surrounding myofibers; CD31^+^ immunostaining (green) identifies endothelial cells as a measure of vascularity. Laminin intensity in the merged images was reduced to enhance visualization of CD31^+^ staining. Yellow arrows identify CD31^+^ staining in merged image. (**C**) Summary values (*n* = 4–5/group) for area occupied by ECs were calculated as: total EC area/number (#) of fibers. (**D**) Summary values (*n* = 4–5/group) for EC-to-myofiber ratio were calculated as: # of EC puncta/# of myofibers. Summary values presented for 14 dpt. Angiogenic factors are increased with TRIM. (**E**) VEGF concentration in whole-muscle TA homogenates (*n* = 5/group) probed for by ELISA. Summary values are means ± SEM; comparisons made vs. WT and vehicle controls by 2-Way ANOVA, *p* < 0.05 = significant. Scale bars = 100 μm.

Angiogenesis has been reported following ion matrix administration in other biological applications [35,36,57,71]. In muscle cross-sections, we evaluated the area of CD31^+^, an established biomarker of endothelial cells that increases with vessel growth [72,73]. This analysis revealed a >2-fold increase in EC area in TA muscles of D2.*mdx* CoO-TRIM mice compared to TA muscles of D2.*mdx* Saline mice (Figure 5A–C, *p* ≤ 0.01), consistent with observations of other ion matrices applied to soft-tissue injuries [36,42,57]. EC area decreased in TA muscles of D2.*mdx* Saline mice compared to TA muscles of WT mouse groups (*p* = 0.01) and increased in TA muscles of D2.*mdx* CoO-TRIM mice compared to TA muscles of WT mouse groups (*p* = 0.05). Additionally, the EC-to-myofiber ratio was greater for TA muscles of D2.*mdx* CoO-TRIM mice (*p* ≤ 0.01) compared to TA muscles of D2.*mdx* Saline mice (Figure 5D). The EC-to-myofiber ratio decreased in TA muscles of D2.*mdx* Saline mice (*p* ≤ 0.01) and was similar in TA muscles of D2.*mdx* CoO-TRIM mice (*p* ≥ 0.99) compared to TA muscles of WT mouse groups, respectively. No differences were observed for EC area (*p* = 0.98) or EC-to-myofiber ratio (*p* = 0.25) in TA muscles of WT mouse groups.

VEGF is a potent mediator of angiogenesis [74,75]. Previous studies have found improvements in diseased muscle function with increased VEGF expression [76,77,78,79]. At 14 dpt, VEGF concentration was ~37% greater (*p* = 0.04) in TA muscles of D2.*mdx* CoO-TRIM mice compared to TA muscles of D2.*mdx* Saline mice (Figure 5E). TA muscles of D2.*mdx* Saline mice had a non-significant increase in VEGF (*p* = 0.13), while TA muscles of D2.*mdx* CoO-TRIM mice increased VEGF (*p* ≤ 0.01) compared to TA muscles of WT mouse groups, respectively. No differences were observed in VEGF concentration (*p* ≥ 0.99) between TA muscles of WT mouse groups.

#### 3.2.4. TA Muscles of D2.*mdx* CoO-TRIM Mice Have Increased IL-6

**Figure 6:**
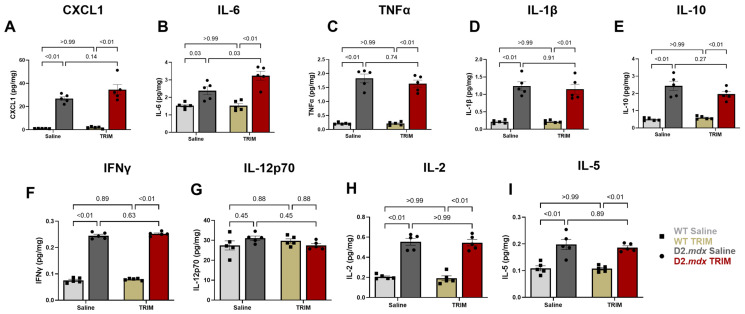
Key inflammatory cytokines from TA muscle homogenates at 14 dpt; (**A**) CXCL1, (**B**) IL-6, (**C**) TNFα, (**D**) IL-1β, (**E**) IL-10, (**F**) IFNγ, (**G**) IL-12p70, (**H**) IL-2, (**I**) IL-5. (*n* = 5/group); Summary values are means ± SEM. Comparisons made vs. WT and vehicle controls by 2-Way ANOVA, *p* < 0.05 = significant.

Early regenerative events in skeletal muscle coincide with inflammatory cell infiltration and cytokine secretion [80]. Investigations of the contribution of inflammation to muscle regeneration indicate involvement of interleukins; in particular, IL-6 can be secreted by intramuscular macrophages, increasing satellite cell proliferation [81,82]. At 14 dpt, IL-6 concentrations were elevated in TA muscles of D2.*mdx* TRIM mice by 36% (Figure 6B, *p* = 0.03) compared to TA muscles of D2.*mdx* Saline mice. TA muscles of D2.*mdx* mice, regardless of treatment, increased CXCL1, TNFα, IL-1β, IL-10, IFNγ, IL-2, and IL-5 compared to TA muscles of WT mouse groups (Figure 6). No differences were observed in any inflammatory cytokine between TA muscles of WT mouse groups.

### 3.3. Experiment 2

#### 3.3.1. TA Muscle Force Improved in D2.*mdx* CoO-TRIM Mice Through 70 Days

**Figure 7:**
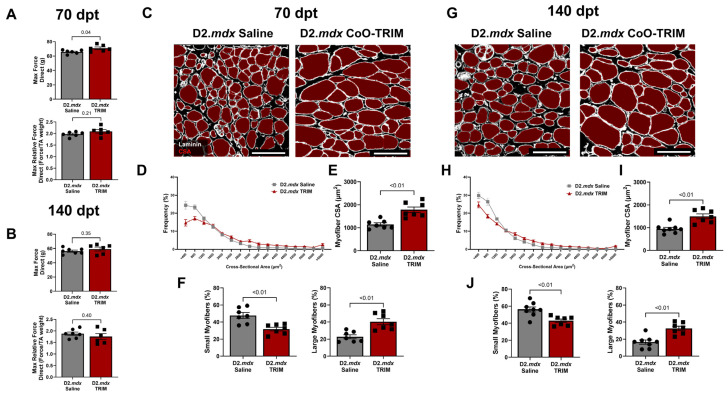
D2.*mdx* treated with CoO-TRIM exhibit greater active force up to 70 dpt. Summary values for max absolute and relative force via direct muscle stimulation at 120 Hz at (**A**) 70 dpt and (**B**) 140 dpt (*n* = 6–7/group). TRIM increases myopathic TA myofiber size. Representative images of TA muscle cross-sections at (**C**) 70 dpt and (**G**) 140 dpt. Laminin (white) identified basal laminae surrounding myofibers (red), which were quantified for CSA. (**D**,**H**) Myofiber CSA frequency distribution at criterion timepoints. Mean myofiber CSA is larger following CoO-TRIM at (**E**) 70 dpt and (**I**) 140 dpt (*n* = 7–8/group). TRIM increases frequency (%) of large myofibers (CSA > 2000 μm^2^) up to 140 dpt and reduces small myofibers (CSA < 800 μm^2^) up to 140 dpt. Summary values comparing small and large myofiber frequency at (**F**) 70 dpt and (**J**) 140 dpt. (*n* = 7–8); summary values are means ± SEM. Comparisons made vs. vehicle controls by two-tailed Student’s *t*-test; *p* < 0.05 = significant. Scale bars = 100 µm.

At 70 dpt, maximal absolute force (g) in TA muscles of D2.*mdx* CoO-TRIM mice improved compared to TA muscles of D2.*mdx* Saline mice (8%, *p* = 0.04), while no changes were observed between groups at 140 dpt (Figure 7A,B). No changes were observed in TA relative force (force/TA weight) at 70 or 140-dpt (Figure 7A,B). TA muscle weight, BW, and TA/BW ratio were unchanged 70 dpt. At 140 dpt, BW was increased (*p* = 0.03) in D2.*mdx* CoO-TRIM mice, with a nonsignificant increase in TA muscle weight (13%, *p* = 0.14) (Supplementary Figure S4).

#### 3.3.2. Myofiber CSA Is Increased in TA Muscles of D2.*mdx* CoO-TRIM Mice

Myofiber CSA was partitioned as in experiment 1. CSA frequency curves were shifted to the right in TA muscles of D2.*mdx* CoO-TRIM mice (Figure 7D,H) at 70 and 140-dpt due to a ~16% reduction in small myofibers (<800 µm^2^, *p* ≤ 0.01) and a ~50% increase in large myofibers (>2000 µm^2^, *p* ≤ 0.01) compared to TA muscles of D2.*mdx* Saline mice (Figure 7F,J). Myofiber CSA increased at 70 and 140 dpt in TA muscles of D2.*mdx* CoO-TRIM by 56% (*p* ≤ 0.01) and 58% (*p* ≤ 0.01) compared to TA muscles of D2.*mdx* Saline mice, respectively (Figure 7E,I).

#### 3.3.3. Central Nucleated Myofibers Are Increased in TA Muscles of D2.*mdx* CoO-TRIM Mice

**Figure 8:**
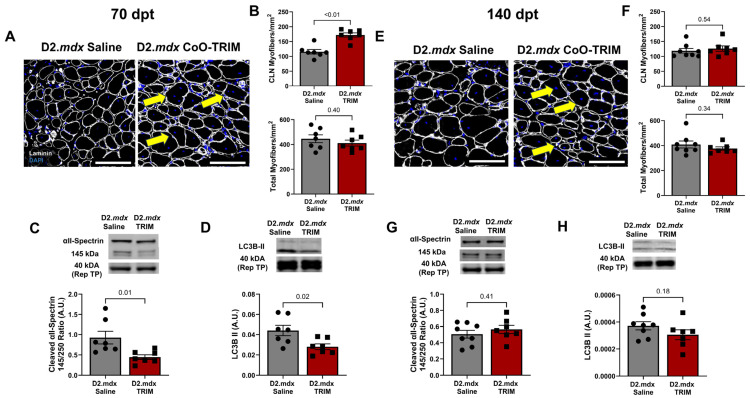
Comparison of CLN in myofibers of WT and D2.*mdx* treated with Saline and TRIM mice. (**A**,**E**) Representative images of TA muscle cross-sections at 70 dpt and 140 dpt. Laminin (white): basal laminae; DAPI (blue): nuclei. Yellow arrows identify position of central nuclei. (**B**,**F**) Summary values for total myofibers with CLN (Top) and total myofibers (CLN^+^ + CLN^−^) (Bottom) normalized to TA muscle cross-section area (mm^2^). Summary values presented for (**B**) 70 dpt and (**F**) 140 dpt. (*n* = 7–8/group); scale bars = 100 µm. Proteolytic activity is altered following CoO-TRIM treatment. (**C**,**G**) Representative immunoblots for αII-Spectrin. The 145 kDa cleavage byproduct was normalized to total protein per lane, represented by the 40 kDa band from the total protein stain, and analyzed as a ratio of the 250 kDa band of αII-Spectrin. Mean densitometric data revealed a significant reduction in cleaved αII-Spectrin abundance in TRIM mice at 70 dpt. There was no difference at 140 dpt. (**D**,**H**) Representative immunoblots of LC3B II and mean densitometric data at (**D**) 70 dpt, revealed that TRIM reduced autophagosome number. No differences were observed at (**H**) 140 dpt. (*n* = 7–8/group); summary values are means ± SEM. Comparisons made vs. vehicle controls by two-tailed Student’s *t*-test; *p* < 0.05 = significant.

Myofiber regeneration in TA muscles of D2.*mdx* CoO-TRIM mice increased by 49% (*p* ≤ 0.01) at 70 dpt (Figure 8A,B), with no difference at 140 dpt (*p* = 0.54) (Figure 8E,F), suggesting that CoO-TRIM may restore the regenerative capacity of myopathic muscle in D2.*mdx* mice up to 70 days [20]. No difference was observed in TA muscles of D2.*mdx* mice for myofiber number at 70 dpt (*p* = 0.40) or 140 dpt (*p* = 0.34) (Figure 8B,F Bottom).

#### 3.3.4. Muscle Proteolysis Is Reduced 70 Days Post-CoO-TRIM Injection

αII-spectrin cleavage byproduct, indicative of calpain activity, was reduced in TA muscles of D2.*mdx* CoO-TRIM mice compared to TA muscles of D2.*mdx* Saline mice (*p* = 0.01) at 70 dpt (Figure 8C). However, by 140 dpt, there was no difference between D2.*mdx* groups (Figure 8G). LC3BII, an indicator of autophagosome formation [70], was reduced in TA muscles of D2.*mdx* CoO-TRIM mice at 70 dpt (*p* = 0.02), while not significant at 140 dpt (*p* = 0.18) (Figure 8D,H) compared to TA muscles of D2.*mdx* Saline mice.

#### 3.3.5. Angiogenesis Is Elevated up to 140 Days Post-CoO-TRIM Injection

**Figure 9:**
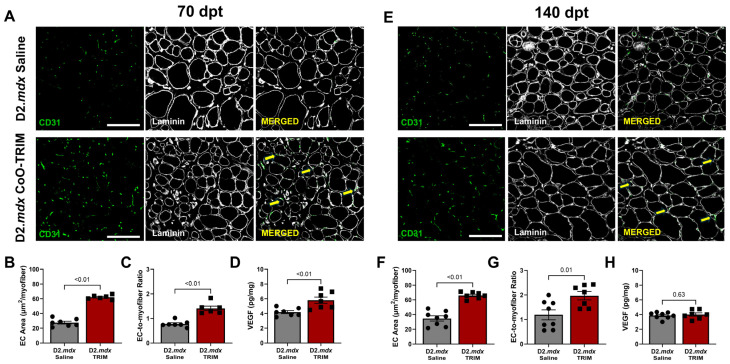
TRIM enhances vascularity. Representative images of TA cross-sections in D2.*mdx* Saline (Top) and D2.*mdx* TRIM (Bottom) mice. (**A**) A 70 dpt and (**E**) 140 dpt. Laminin+ immunostaining (white) identified basal laminae surrounding myofibers; CD31^+^ immunostaining (green) identifies endothelial cells as a measure of vascularity. Laminin intensity in the merged images was reduced to enhance visualization of CD31^+^ staining. Yellow arrows identify CD31^+^ staining in merged image. Summary values (*n* = 7–8/group) for area occupied by ECs were calculated as: total EC area/number (#) of myofibers. Summary values presented for (**B**) 70 dpt and (**F**) 140 dpt. Summary values (*n* = 7–8/group) for EC-to-myofiber ratio were calculated as: # of EC puncta/# of myofibers, presented for (**C**) 70 dpt and (**G**) 140 dpt. Angiogenic factors are increased with TRIM. VEGF concentration in whole muscle TA homogenates probed for by ELISA at (**D**) 70 dpt and (**H**) 140 dpt. (*n* = 7–8/group); summary values are means ± SEM. Comparisons made vs. vehicle controls by two-tailed Student’s *t*-test; *p* < 0.05 = significant. Scale bars = 100 μm.

In muscle cross-sections, we evaluated the area of CD31^+^, an established biomarker of endothelial cells that increases with vessel growth [72,73]. This analysis revealed a >2-fold increase in EC area in TA muscles of D2.*mdx* TRIM mice at 70 (*p* ≤ 0.01) and 140-dpt (*p* ≤ 0.01) compared to TA muscles of D2.*mdx* Saline mice (Figure 9A,B,E,F), consistent with observations of other ion matrices applied to soft-tissue injuries as mentioned above [36,42,83]. Moreover, the EC-to-myofiber ratio increased in TA muscles of D2.*mdx* TRIM mice at 70 (~80%, *p* ≤ 0.01) and 140 dpt (~66%, *p* ≤ 0.01) compared to TA muscles of D2.*mdx* Saline mice (Figure 9C,G).

#### 3.3.6. VEGF Is Increased in TA Muscles of D2.*mdx* CoO-TRIM Mice Through 70 Days

At 70 dpt, VEGF concentration was ~39% greater (*p* ≤ 0.01) in TA muscles of D2.*mdx* CoO-TRIM mice compared to TA muscles of D2.*mdx* Saline (Figure 9D). At 140 dpt, there were no differences between TA muscles of D2.*mdx* groups (Figure 9H).

#### 3.3.7. Inflammatory Cytokines from TA Muscles of D2.*mdx* Mice Through 140 Days

No differences were observed in any cytokine except for IL-5 (*p* ≤ 0.01) at 70 days nor 140 days in TA muscles of D2.*mdx* CoO-TRIM mice compared to TA muscles of D2.*mdx* Saline mice (Supplementary Figure S5A,B).

## 4. Discussion

Borate-based glasses injected as micron-sized particles are promising inorganic biomaterials for restoring damaged skeletal muscle and stimulating VEGF production following injury [36,57]. The borophosphate glass formulation of TRIM used in the present experiments was designed to release biologically active concentrations of borate, phosphate, and cobalt ions to promote myofiber regeneration and angiogenesis. While previous studies investigated the therapeutic efficacy of an inorganic biomaterial in the context of skeletal muscle injury [36,57], the present experiments are the first to apply these materials to skeletal muscle disease. We focused on answering two questions: (1) Does CoO-TRIM restore skeletal muscle structure and function in myopathic mice (Experiment 1)? (2) How persistent are the effects of CoO-TRIM in myopathic muscle (Experiment 2)? The TA muscle in the mouse hindlimb was studied owing to its superficial location and accessibility for in situ, isometric force measurements [58,84]. Herein we report that when TA muscles of D2.*mdx* CoO-TRIM mice were compared to TA muscles of D2.*mdx* Saline mice: (1) max isometric force was improved up to 70 dpt, (2) myofiber regeneration in myopathic muscle was improved, (3) myofiber sizes were larger, (4) proteolytic markers were reduced through 70 dpt, (5) muscle vascularity was enhanced up to 140 dpt, (6) VEGF protein concentration increased through 70 dpt, and (7) IL-6 was greater at 14 dpt while IL-5 was lower at 140 dpt. In contrast, no differences were observed in any of these measures in WT CoO-TRIM mice compared to WT Saline mice.

### 4.1. Properties of Inorganic Biomaterials During Restoration of Skeletal Muscle

Silicate glasses have been studied for their bone regeneration and wound healing properties [31,33,61,85,86]. The use of borate-based glass has increased due to its rapid degradation and increased bioactivity compared to traditional silica-based materials (45S5 and related glasses, nano-silicates) [27,29,37,87]. Boron plays an essential role in angiogenesis and regeneration [88,89,90] but the rapid dissolution of borate glass can promote alkaline conditions and toxicity [91]. To avoid these effects, glass compositions can be modified to control the degradation rate and pH conditions [33,35]. For example, additions of network oxides, like P_2_O_5_ and Al_2_O_3_, and transition metal oxides, like CoO and CuO, modify the borate glass structure decreasing degradation rates to provide a therapeutic environment for regeneration and so enhance their long-term biological actions [36,42,92,93,94]. Borophosphate-based glasses such as the TRIM developed for soft-tissue repair have controlled kinetics of degradation and ion solubility [35,37,39,95]. The present findings demonstrate our novel CoO-TRIM composition concentrates P_2_O_5_ and CoO in the precipitated amorphous calcium polyphosphate (Table 1) for continued gradual release, consistent with stimulating regeneration while maintaining a pH-neutral environment.

The single-pass flow through (SPFT) test was developed to screen the dissolution behavior of materials exposed to a well-established physiological solution (SBF) under the dynamic conditions expected in vivo. Nevertheless, the effects on the quantitative dissolution characteristics of the CoO-TRIM particles in the SPFT test are expected to be different from those experienced in vivo. For example, the surface adsorption of proteins reduces ion release rates from bioactive glasses in cell culture media relative to serum-free solutions [96]. Similar processes are expected to affect the in vivo dissolution rates in the present study. Increasing the [surface area (SA)] of glass particles relative to the solution volume (V) increases ion release rates [97]. Based on the relative volumes of the SPFT reaction cell (16 mL) and the TA muscle compartments (0.058 mL) and the mass of glass particles used in both experiments (200 mg and 250 mg, respectively), the relative SA-to-V ratio in the SPFT test is greater; therefore, faster ion release rates would be expected in this test. Finally, ion release rates also depend on the type of buffer used, with faster release rates associated with TRIS-buffered SBF compared to non-buffered SBF [98], which may also contribute to faster ion release rates in the SPFT than in vivo. Given these factors, the transient acidification noted in the SPFT results may not be occurring under the expected slower reaction conditions in vivo.

Conventional bioactive glasses are designed to form hydroxyapatite (HA) layers after implantation into biological environments [22,31,99]. While beneficial for bone regeneration, HA formation can promote myofiber calcification (i.e., myositis ossificans), making traditional glass compositions undesirable for soft-tissue applications. HA forms under the alkaline conditions created by the dissolution of conventional bioactive glass compositions in the presence of phosphate-containing solutions, whereas the pH-neutral borophosphate TRIM used here inhibits HA formation [35,37,100,101]. First, when phosphate ions are released from borophosphate glass, phosphoric acid buffers the alkaline effects from the concomitant release of the alkali ions, neutralizing local pH change, creating conditions that favor the precipitation of amorphous calcium phosphates [101,102,103]. Second, pyrophosphate anions are released from the reacting borophosphate glasses, and these are then concentrated in the precipitating amorphous polyphosphate phase, blocking HA crystallization [66,67]. Moreover, the incorporation of cobalt ions in the amorphous calcium polyphosphate also inhibits HA formation [40,46]. The slow release of cobalt ions (Figure 2D) and the formation of amorphous calcium polyphosphate material particles (Figure 2F) indicate that CoO-TRIM controls ion release when injected into myopathic skeletal muscle, maintains pH-neutrality, and inhibits HA formation while stimulating myofiber regeneration and angiogenesis. Co concentrations were evaluated during the dynamic conditions of the SBFT, which were designed to mimic in vivo circulation. Thus, Co ions would be released within a physiologically relevant range as CoO-TRIM particles degrade. Because the physiological bicarbonate buffering system may slow degradation compared to the TRIS buffering of SBF, Co ion concentrations in vivo may be slightly lower than in dynamic SBFT tests. Future studies are necessary to analyze in vivo ion concentrations over time.

### 4.2. Skeletal Muscle Function and Structure Following CoO-TRIM Injection

The D2.*mdx* mouse exhibits severe skeletal muscle pathology characterized by progressive atrophy and weakness, recapitulating a severe myopathic phenotype [49,50]. At 7 months of age, D2.*mdx* hindlimb muscles present significant reductions in myofiber size and 50% loss of strength [54]. Moreover, chronic myofiber degeneration exhausts the regenerative capacity, culminating in satellite cell depletion [19,20,55,104], tissue necrosis and impaired muscle function [11,12]. While borate TRIMs can promote soft tissue regeneration [36,42,57], this is the first study to investigate the functional recovery of diseased skeletal muscle following the introduction of an inorganic biomaterial. The present findings show that skeletal muscle structure and function were improved in TA muscles of D2.*mdx* mice up to 70 days following a single injection of CoO-TRIM, while no changes were observed in TA muscles of healthy WT mice. Furthermore, myofiber regeneration (based on CLN) and myofiber size were greater in TA muscles of D2.*mdx* CoO-TRIM mice compared to TA muscles of D2.*mdx* Saline mice without accompanying changes in fibrotic tissue deposition. Behavioral observations only suggested normal scratch pad engagement from WT and CoO-TRIM-treated D2.*mdx* mice as early as 14 days following injection, while Saline-treated D2.*mdx* mice showed no scratch pad engagement.

At 14 dpt, we show TA muscles of D2.*mdx* CoO-TRIM mice improved functional capacity as relative force was increased, yet muscle weight was unchanged. Although relative force was no longer elevated at 70 days, TA muscles of D2.*mdx* CoO-TRIM mice maintained the ability to produce greater total force compared to TA muscles of D2.*mdx* Saline mice with increased absolute values, owing to the synchronous improvements in myofiber size and regeneration. Interestingly, TA muscles of D2.*mdx* Saline mice significantly increased myofiber number compared to WT mice groups, while TA muscles of D2.*mdx* CoO-TRIM mice were similar in number to WT mouse groups. The increased myofiber number is consistent with pseudo-hyperplasia driven by ongoing degeneration, myofiber splitting, and incomplete regeneration [105,106,107]. We show CoO-TRIM normalized myofiber number to levels observed in TA muscles of WT mouse groups with increased myofiber size, regeneration, and force production, indicating reduced pathological remodeling and improved myofiber maturation. These findings suggest TA muscles of D2.*mdx* CoO-TRIM mice improved muscle quality and functional integrity rather than promoting compensatory increases in myofiber number.

As myopathy progresses, damaged myofibers activate calpains and autophagy, initiating widespread proteolysis [68,108]. Although necessary following acute muscle injury for breakdown and clearance of necrotic tissue, excessive activation under myopathic conditions contributes to muscle cell death and increased pathology [68,109,110]. Our results following CoO-TRIM injection show significantly reduced αII-spectrin cleavage, indicative of calpain activity, up to 70 dpt, consistent with previous reports that calpain inhibition in *mdx* mice promotes myofiber size and function [16,111]. In addition, the protein marker LC3II, indicative of autophagosome formation, was also reduced following CoO-TRIM treatment up to 70 dpt [112]. Therefore, we suggest that increased myofiber size and contractile force following CoO-TRIM injection reflect a reduction in proteolytic activity indicators, further supporting muscle regeneration.

### 4.3. Angiogenesis and VEGF Are Increased Following CoO-TRIM Injection

Vascular dysfunction is a prominent contributor to myopathic conditions characterized by a reduction in endothelial cell density, impaired angiogenesis, perturbations in vasodilation, and insufficient blood supply to degenerating muscles [15,113,114,115,116]. Impaired angiogenesis further contributes to delayed or incomplete regeneration, suggesting a requirement for vascularization in forming functional myofibers [116,117,118]. Moreover, reduced muscle damage in myopathic mice has been associated with enhanced angiogenesis and overexpression of VEGF [76,77,78,79]. Our work complements earlier studies demonstrating that ions released from CoO-TRIM can stimulate VEGF production and subsequent angiogenesis [35,36,42,57], with cobalt capable of acting as a hypoxia mimetic through binding to HIF-1α and inducing its translocation to the nucleus, thereby activating VEGF release [63,119]. The short half-life of VEGF (<30 min) and off-target effects of its systemic administration have prevented its use in the clinic, highlighting the need for a therapy capable of local VEGF stimulation with subsequent angiogenic effects. By evaluating muscle vascularity (CD31^+^ area and EC-to-myofiber ratio) and angiogenic growth factors, we find more microvessels and increased VEGF concentration in myopathic muscle following CoO-TRIM treatment. An additional benefit of increased microvascular perfusion is more effective delivery of systemic therapeutics.

At 14 and 70-dpt, D2.*mdx* CoO-TRIM mice, but not WT CoO-TRIM mice, exhibited increased VEGF in the TA muscle compared to D2.*mdx* Saline mice, concomitant with improvements in muscle function, myofiber size, and vascular area. At 140 dpt, the vascular area remained elevated; however, VEGF was no longer increased. At this later time, maximal isometric force was similar to D2.*mdx* Saline mice, and myofiber sizes (CSA) were smaller compared to earlier timepoints. These findings demonstrate that an increase in vascular area alone is insufficient to restore muscle structure and function in myopathy. For example, at 140 dpt, CD31^+^ labeling was elevated despite no differences in VEGF or maximal force from TA muscles of D2.*mdx* Saline mice. These data suggest VEGF may promote independent contributions to diseased muscle function beyond its role in angiogenesis. VEGF promotes proliferation and maturation of satellite cells that may contribute to restoring the impaired regenerative capacity [120,121,122,123], thereby improving myopathic skeletal muscle structure and function. Concomitant with a rise in VEGF in TA muscles of D2.*mdx* mice with CoO-TRIM, IL-6 was increased compared to TA muscles of D2.*mdx* Saline mice. Notably, CoO-TRIM did not elicit an inflammatory response in healthy WT muscle, whereas in the context of preexisting inflammation in the D2.*mdx* mouse, CoO-TRIM treatment was associated with functional and structural benefits, including increased VEGF expression. These findings suggest CoO-TRIM-mediated improvements to myopathic skeletal muscle may be immune cell-dependent and require a preexisting inflammatory response [57]. Although IL-6 was transiently elevated at 14 dpt following CoO-TRIM treatment, overall inflammation remained increased in the D2.*mdx* mouse compared to WT mice, independent of treatment, with IL-6, TNF-α, and IL-10 known as potent stimulators of VEGF [124,125,126]. Thus, we suggest CoO-TRIM may act by amplifying acute inflammatory responses, thereby promoting VEGF secretion and downstream angiogenic and regenerative effects without causing inflammation in healthy skeletal muscle or exacerbating chronic inflammation. Based on the resolution of functional restoration, VEGF increase, indicators of proteolytic reduction, and decreased average myofiber size compared to 14 dpt, we suggest CoO-doped BPBG particles undergo complete degradation in skeletal muscle between 70 and 140 dpt. Future pharmacokinetic studies are necessary to identify the precise timing of complete particle degradation and metabolism in vivo.

## 5. Conclusions

Inorganic biomaterials have been used to stimulate regeneration in hard tissues, wound healing, and muscle injury. The present study shows that following administration of an inorganic biomaterial, muscle structure, function, and angiogenesis are improved in the context of myopathy but not in WT mice. These results support our hypothesis that isometric force and myofiber CSA in skeletal muscle of D2.*mdx* mice would be improved following CoO-TRIM treatment compared to D2.*mdx* Saline mice. We conclude that muscle structure, function, and angiogenesis are improved in D2.*mdx* mice following a single injection of CoO-TRIM as early as 14 dpt and beneficial responses persist for 70 dpt.

## 6. Patents

The work described in this article is covered by patent application no. WO2023034523A1 [127]. A.B.M., S.S.S., and R.K.B. are co-inventors of CoO-TRIM.

## Figures and Tables

**Table 1:** Molar concentrations of CoO-TRIM particles. Values are mean ± SEM molar compositions of CoO-TRIM before and after 7 d single-pass flow through (SPFT) test with 37 °C simulated body fluid (SBF).

Ion	Before SPFT (mol)	After SPFT (mol)
Na_2_O	14.4 ± 0.2	1.5 ± 0.0
CaO	21.8 ± 0.2	55.8 ± 0.2
MgO	0.0 ± 0.0	4.8 ± 0.0
CoO	4.0 ± 0.0	10.7 ± 0.0
B_2_O_3_	40.5 ± 0.6	1.8 ± 0.0
P_2_O_5_	19.3 ± 0.3	25.3 ± 0.2

## Data Availability

The data that support the findings of this study are available from the corresponding author upon reasonable request.

## Supplementary Materials

The following supporting information can be downloaded at: https://www.mdpi.com/article/10.3390/jfb17030155/s1, Figure S1: Representative images of tibialis anterior (TA) and extensor digitorum longus (EDL) muscles (within white ovals) injected with saline or Eu-TRIM. Note while the TA muscle was injected, TRIM diffuses to the EDL as evidenced by pink fluorescent signal (yellow arrows), Scale bars = 2 mm; Figure S2: TRIM does not affect fibrosis in D2.*mdx* mice. At 14 dpt, fibrotic tissue deposition is unchanged following TRIM treatment in D2.*mdx* TA muscles. (*n* = 5); Summary values are means ± SEM. TRIM vs. vehicle (Saline) comparisons performed with two-tailed Student’s *t* test, *p* < 0.05 = significant. Scale bars = 200 µm; Figure S3: Summary values for percentage of CLN^+^ myofibers in TA muscle cross-section, analyzed as CLN^+^ myofibers/total myofibers × 100. (Left) 14 dpt (*n* = 4–5/group), (Middle) 70 dpt (*n* = 7), Right) 140 dpt (*n* = 7–8). Summary values are means ± SEM. 14 dpt, TRIM comparisons vs. WT and vehicle (Saline) controls performed by 2-Way ANOVA and, *p* < 0.05 = significant. 70- and 140 dpt, TRIM vs. vehicle (Saline) comparisons performed with two-tailed Student’s *t* test, *p* < 0.05 = significant; Figure S4: D2.*mdx* mice with TA muscles injected with CoO-TRIM had increased body weight at 140 days, but no changes in TA weight or muscle-to-body weight ratio. (*n* = 6–7); Summary values are means ± SEM. TRIM vs. vehicle (Saline) performed with two-tailed Student’s *t* test, *p* < 0.05 = significant; Figure S5A,B: Inflammatory cytokines from TA homogenates at Top row: 70 dpt and Bottom row: 140 dpt. Supplementary 5A; (A,F) CXCL1, (B,G) IL-6, (C,H) TNFα, (D,I) IL-1β, (E,J) IL-10. Supplementary 5B; (A,E) IFNγ, (B,F) IL-12p70, (C,G) IL-2, (D,H) IL-5. (*n* = 7–8/group); Summary values are means ± SEM. TRIM vs. vehicle (Saline) comparisons performed with two-tailed Student’s *t* test, *p* < 0.05 = significant.
