# Supplementary material for: A Borophosphate Glass Doped with Cobalt Oxide Improves Skeletal Muscle Structure and Function in Myopathic Mice
Source: J Funct Biomater. 2026 Mar 20;17(3):155. doi: 10.3390/jfb17030155 (PMC13028039; doi:10.3390/jfb17030155)
Supplement: Supplementary file 1 [file jfb-17-00155-s001.zip › jfb-4175736-supplementary.pdf]

Supplementary Material

Supplementary 1

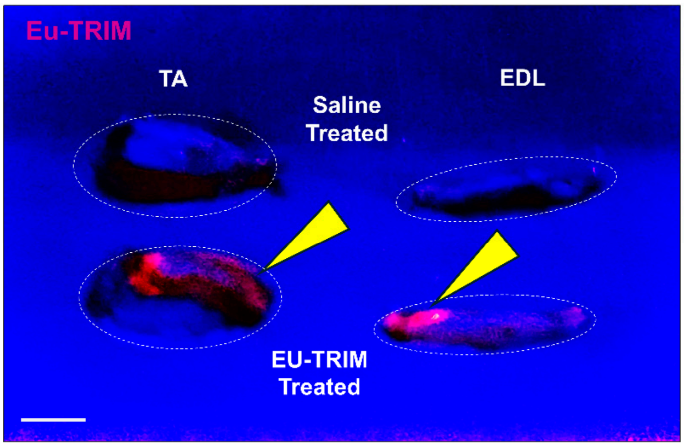

Supplementary Figure S1. Representative images of tibialis anterior (TA) and extensor digitorum longus (EDL) muscles (within white ovals) injected with saline or Eu-TRIM. Note while the TA muscle was injected, TRIM diffuses to the EDL as evidenced by pink fluorescent signal (yellow arrows), Scale bars = 2 mm.

Supplementary 2

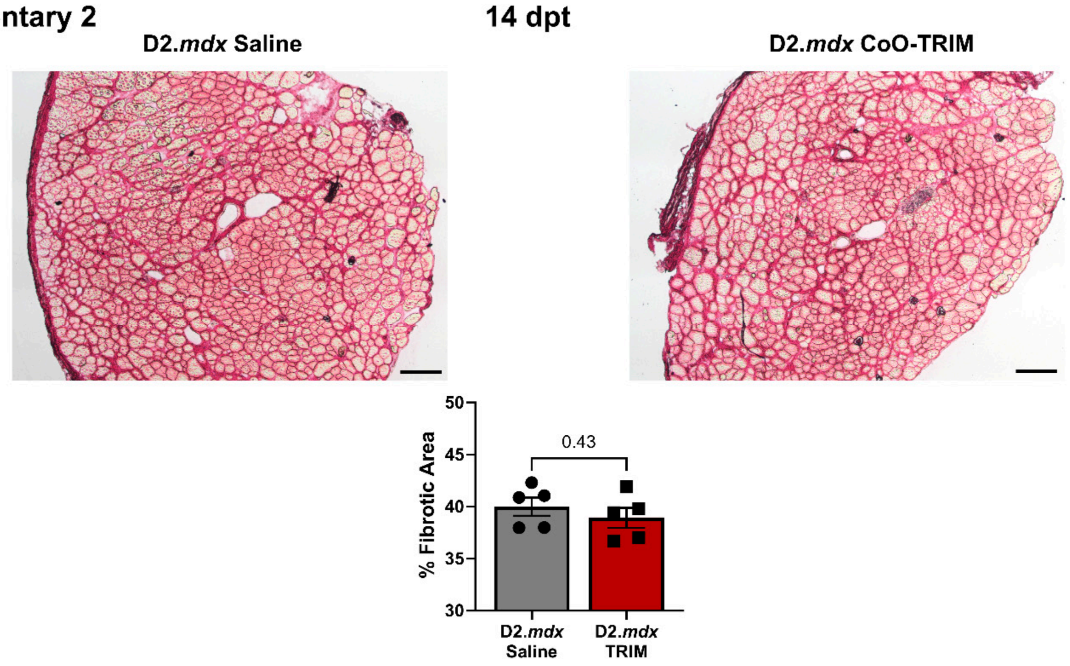

Supplementary Figure S2. TRIM does not affect fibrosis in D2.*mdx* mice. At 14 dpt, fibrotic tissue deposition is unchanged following TRIM treatment in D2.*mdx* TA muscles. (n=5); Summary values are means ± SEM. TRIM vs. vehicle (Saline) comparisons performed with two-tailed Student's *t* test, $p<0.05$ = significant. Scale bars = 200 µm.

Supplementary 3

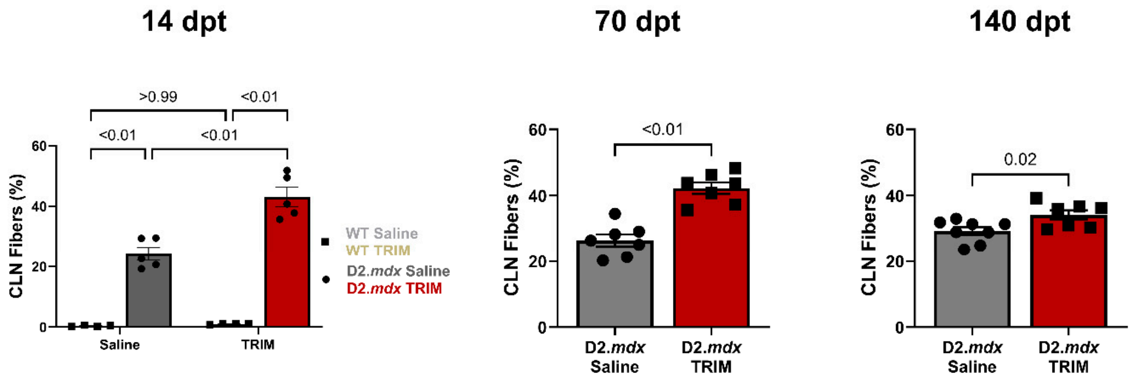

Supplementary Figure S3. Summary values for percentage of CLN⁺ myofibers in TA muscle cross-section, analyzed as CLN⁺ myofibers/total myofibers x 100. Left) 14 dpt (n=4-5/group), Middle) 70 dpt (n=7), Right) 140 dpt (n=7-8). Summary values are means ± SEM. 14 dpt, TRIM comparisons vs. WT and vehicle (Saline) controls performed by 2-Way ANOVA and , $p<0.05$ = significant. 70- and 140 dpt, TRIM vs. vehicle (Saline) comparisons performed with two-tailed Student's t test, $p<0.05$ = significant.

Supplementary 4

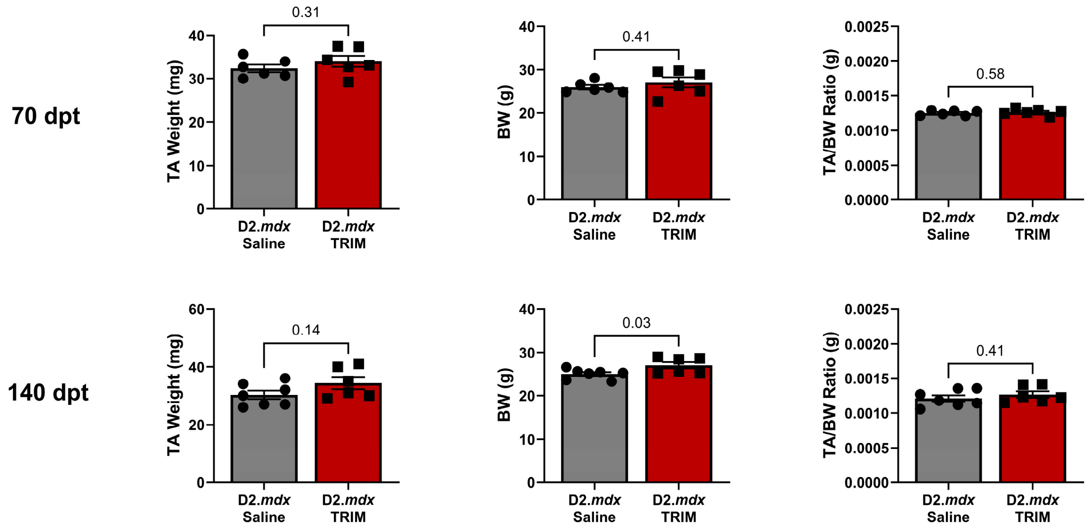

Supplementary Figure S4. D2.*mdx* mice with TA muscles injected with CoO-TRIM had increased body weight at 140 days, but no changes in TA weight or muscle-to-body weight ratio. (n=6-7); Summary values are means ± SEM. TRIM vs. vehicle (Saline) performed with two-tailed Student's *t* test, $p<0.05$ = significant.

Supplementary 5A

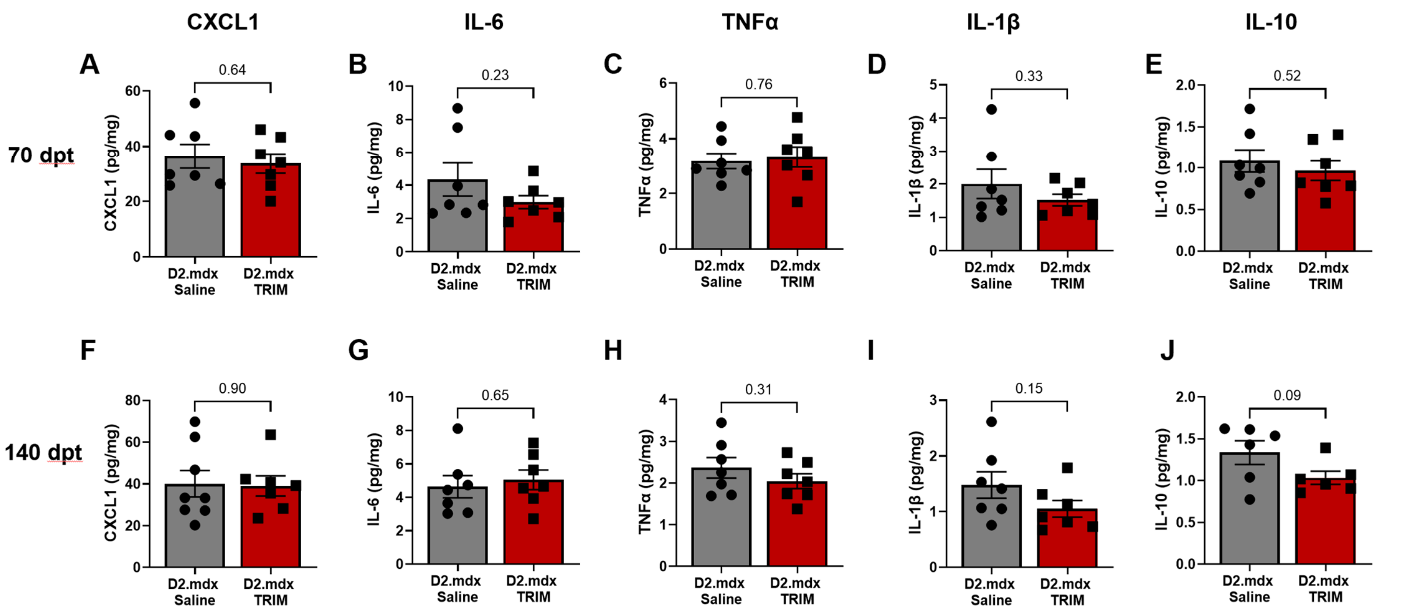

Supplementary 5B

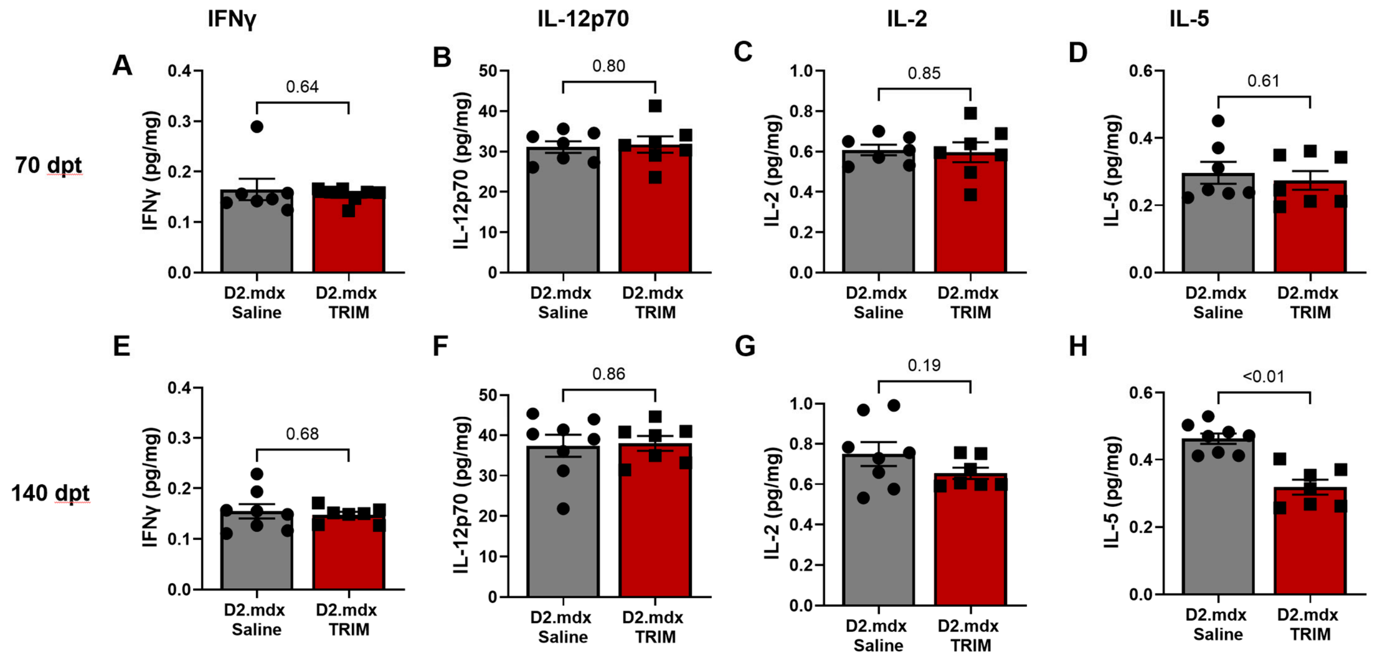

Supplementary Figure S5. Inflammatory cytokines from TA homogenates at Top row: 70 dpt and Bottom row: 140 dpt. Supplementary 5A; A,F) CXCL1, B,G) IL-6, C,H) TNFα, D,I) IL-1β, E,J) IL-10. Supplementary 5B; A,E) IFNγ, B,F) IL-12p70, C,G) IL-2, D,H) IL-5. (n=7-8/group); Summary values are means ± SEM. TRIM vs. vehicle (Saline) comparisons performed with two-tailed Student's *t* test, $p<0.05$ = significant.
